# Sex differences in the association between epicardial adipose tissue volume and left atrial volume index

**DOI:** 10.1186/s12872-023-03569-1

**Published:** 2024-01-13

**Authors:** Satoshi Yamaguchi, Minetaka Maeda, Kageyuki Oba, Gulinu Maimaituxun, Osamu Arasaki, Shusuke Yagi, Kenya Kusunose, Takeshi Soeki, Hirotsugu Yamada, Daiju Fukuda, Hiroaki Masuzaki, Masataka Sata, Michio Shimabukuro

**Affiliations:** 1 Department of Diabetes, Endocrinology and Metabolism, Fukushima Medical University School of Medicine, 1 Hikarigaoka, Fukushima, 960-1247 Japan; 2 Department of Cardiology, Nakagami Hospital, 610 Noborikawa, Okinawa, 904-2195 Japan; 3 Department of Cardiology, Yuuai Medical Center, 50-5 Yone, Tomishiro, 901-0224 Okinawa Japan; 4 Department of Cardiovascular Medicine, Tokushima University Hospital, 2-50-1 Kuramoto-cho, Tokushima, 770-0042 Japan; 5 Department of Cardiovascular Medicine, Nephrology, and Neurology, Graduate School of Medicine, University of the Ryukyus, 207 Nishihara-cho, Okinawa, 903-0215 Japan; 6 Department of Cardiovascular Medicine, Osaka Metropolitan University Graduate School of Medicine, 1-4-3 Asahi-machi, Abeno-ku, Osaka, 545-8585 Japan; 7 Division of Endocrinology, Diabetes and Metabolism, Hematology, Rheumatology (Second Department of Internal Medicine), Graduate School of Medicine, University of the Ryukyus, 207 Nishihara-cho, Okinawa, 903-0215 Japan

**Keywords:** Epicardial adipose tissue, Left atrial volume index, Sex difference, Atrial fibrillation

## Abstract

**Background:**

Sex disparities in the association between epicardial adipose tissue volume (EATV) and cardiovascular disease have been reported. The sex-dependent effects of EATV on left atrial (LA) size have not been elucidated.

**Methods:**

Consecutive 247 subjects (median 65 [interquartile range 57, 75] years; 67% of men) who underwent multi-detector computed tomography without significant coronary artery disease or moderate to severe valvular disease were divided into two groups: patients with sinus rhythm (SR) or atrial fibrillation (AF). Sex differences in the association between the EATV index (EATVI) (mL/m^2^) and LA volume index (LAVI) in 63 SR (28 men and 35 women) and 184 AF (137 men and 47 women) patients were evaluated using univariate and multivariate regression analyses.

**Results:**

In overall that includes both men and women, the relationship between EATVI and LAVI was not significantly correlated for patients with SR and AF. The relationship between EATVI and LAVI differed between men and women in both SR and AF groups. In SR patients, there was a positive relationship between EATVI and LAVI in men, but not in women. In contrast, in patients with AF, a negative relationship was found between EATVI and LAVI in women, whereas no association was found in men.

**Conclusions:**

We evaluated sex differences in the association between EATVI and LAVI in patients with either SR or AF, and found a positive relationship in men with SR and a negative relationship in women with AF. This is the first report to evaluate sex differences in the relationship between EATVI and LAVI, suggesting that EAT may play a role, at least in part, in sex differences in the etiology of AF.

## Introduction

Epicardial adipose tissue (EAT) is a metabolically active tissue that structurally neighbors the myocardium and the coronary arteries [1, 2]. EAT volume (EATV), as well as visceral adipose tissue, increases in obese patients and correlates with the presence and incidence of coronary artery disease (CAD) independent of traditional CAD risk factors [3, 4]. EATV has been reported to be an independent predictor of left ventricular (LV) remodeling and LV diastolic dysfunction in patients with CAD or metabolic syndrome [5–7]. Excessive accumulation of EAT might have a paracrine or mechanical burden on the coronary microcirculation and myocardium [5].

Previously, we found that the EATV index (EAVTI; EATVI = EATV/body surface area, mL/m^2^) was strongly associated with the prevalence of paroxysmal atrial fibrillation (PAF) and persistent atrial fibrillation (PeAF) in a model adjusted for known atrial fibrillation (AF) risk factors [8]. An association between the EAT and AF prevalence has also been reported [8, 9]. Mahabadi et al. reported that EATV was significantly associated with prevalent AF, independent of AF risk factors; however, this effect was considerably reduced when corrected for left atrial (LA) size [9].

Sex disparities in the association between EATV and cardiovascular disease have been reported. We previously reported that EATV was a discriminator in men but not in women in patients with CAD [10] or in those who underwent coronary artery bypass graft surgery [11]. Until now, the sex-dependent impact of EATV on LA size has not been elucidated. In addition, there are no reports regarding sex disparities in the association between EATV and AF.

This study evaluated sex differences in the association between EATVI and LA volume index (LAVI) in patients with sinus rhythm (SR) or AF.

## Materials and methods

### Participants and data collection

**Fig. 1:**
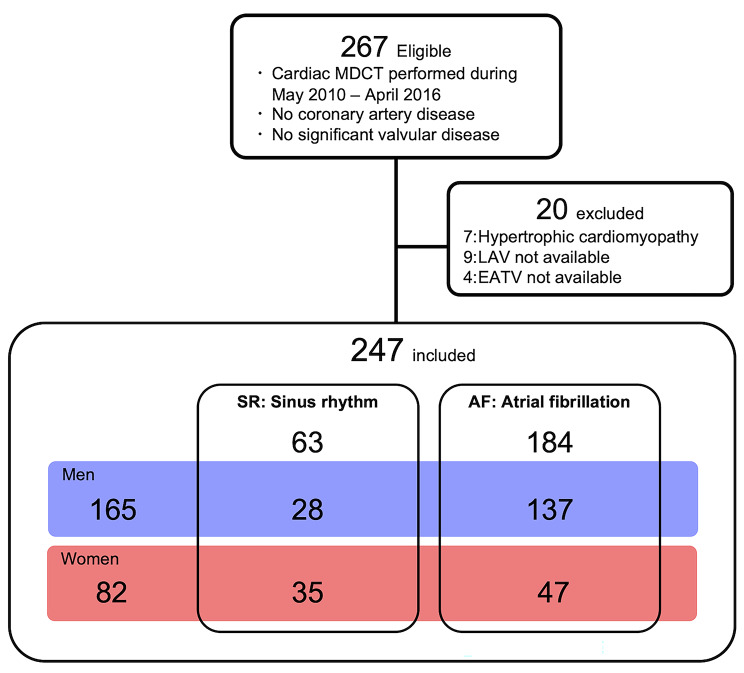
Flowchart of patient recruitment. MDCT: multi-detector row computed tomography; LAV: left atrial volume; EATV: epicardial adipose tissue volume. See the detail in the text

We retrospectively reviewed 267 consecutive Japanese patients who had undergone multi-detector cardiac computed tomography (MDCT) between May 2010 and April 2016 at Tomishiro Central Hospital, Okinawa, Japan, or at the Tokushima University Hospital, Tokushima, Japan (Fig. 1, flowchart of patient recruitment). The subjects had undergone MDCT if they had had symptoms suggestive of symptomatic or asymptomatic coronary artery disease (CAD) in a moderate-to-high CAD risk category [12] or dyspnea suggestive of paroxysmal or chronic AF. The major exclusion criteria were as follows: LV ejection fraction (LVEF) < 50%; serum creatinine levels > 1.5 mg/dL; CAD, if ≥ 1 major coronary artery branch stenosis ≥ 50%; class III or IV heart failure; iodine-based allergy; overt liver disease; hypothyroidism; and moderate to severe valvular disease. Of the 267, 20 patients were excluded because of hypertrophic cardiomyopathy (n = 7), unmeasured LA volume (n = 9), and unmeasured EATV (n = 4). The remaining 247 patients (165 men and 82 women) were enrolled in the full analysis set. The patients were divided into the SR and AF groups. Clinical data, including CT and echocardiographic datasets, were collected from the electrical records by MM, KO, and GM, and anonymous datasets were analyzed offline by SY and MSh.

### Ethics approval and consent to participate

The ethical committees approved the present study (Fukushima Medical University #2019 − 182, Tomishiro Central Hospital R01R027). The need for informed consent was waived by the Ethics Committee/Institutional Review Board of Fukushima Medical University and Tomishiro Central Hospital because of the retrospective nature of the study and the lack of direct patient contact or intervention. All methods were carried out in accordance with the declaration of Helsinki.

### Multi-detector computed tomography

Cardiac CT was performed using a 320-slice CT scanner (Aquilion One; Toshiba Medical Systems, Tokyo, Japan) with 0.275-ms rotation and 0.5/320/0.25 collimation. CT images were acquired using a retrospective, nonhelical electrocardiogram-triggered acquisition mode protocol (tube voltage, 120 kV; tube current, 450 mA × 5 ms) with a thickness of 5-mm slices [10, 13, 14]. All reconstructed CT image data were transferred to an offline workstation (Synapse Vincent, ver. 4.4, Fuji Film, Tokyo, Japan). For measurement of EATV, the pericardium was manually traced in each trans-axial slice, and then automated processing detected the voxels with a density range of -190 to -30 Hounsfield units beneath the pericardium. The cranial and caudal borders of the epicardial adipose tissue were set at the edge of the left pulmonary artery origin and the left ventricular apex.

### Transthoracic echocardiography

Experienced technicians performed comprehensive transthoracic echocardiography according to the American Society of Echocardiography guidelines [15, 16]. Under the guidance of staff cardiologists, the left atrium was traced in the apical 4-chamber and 2-chamber views at the mitral valve level in end-systole, with care taken to exclude the left atrial appendage and pulmonary veins. LA volume (mL) was calculated using the biplane area-length method, and LAVI (mL/m^2^) was divided by body surface area [16]. LVEF was measured using the modified Simpson’s biplane method. Transmitral flow (TMF) velocity was recorded from the apical long-axis or 4-chamber view, and the peak early diastolic (E) TMF velocities were measured. The mitral annular motion velocity pattern was recorded using the apical 4-chamber view with the sample volume located at the lateral or septal side of the mitral annulus using pulsed tissue Doppler echocardiography. The mean peak early diastolic mitral annular velocity (E’) was measured on the septal and lateral sides, and the E to E’ ratio (E/E’) was calculated as a marker of LV filling pressure, as described previously [14].

### Statistical analysis

Continuous variables were expressed as mean ± standard deviation for normal distribution and median [25%, 75%] for non-normal distribution. Categorical variables were expressed as the number of patients with percentages. The t-test and Mann-Whitney U test were used for continuous variables, and Fisher exact test for categorical variables for two-group comparisons. Our patients were a different population, with an SR group suggesting symptomatic or asymptomatic CAD and an AF group with dyspnea suggesting paroxysmal or chronic atrial fibrillation. For this reason, inter-group comparisons were not made between the SR and AF groups but only intra-group comparisons were made. Univariate and multivariate linear regression models were performed to determine factors associated with left atrial volume index in the overall, SR, and AF groups after being divided into men and women. For multivariate analysis, the selected variables were Model 1 (age, BMI, men gender, and EATVI) and Model 2 (Model 1 + LVEF, antihypertensive drug). Univariate and multivariate linear regression models to estimate LAVI were also performed in the following subgroups: ≤65 and > 65-year-old, BMI ≤ 25 and > 25 kg/m^2^, diabetes mellitus yes or no, and hypertension yes or no. Statistical analysis were done by using Exploratory 6.9.4.1 (Exploratory Inc., Mill Valley, CA, USA), Prism 9.3.1 (GraphPad Software Inc., La Jolla, CA, USA), and R 4.0.2 (R Foundation for Statistical Computing, Vienna, Austria). All statistical tests were two-tailed, and statistical significance was set at P < 0.05.

## Results

### General characteristics

#### Overall

General characteristics were shown in both men and women (Table 1). Overall, 247 patients had a mean age of 65 years; 67% were men, 26% had SR, and 74% had AF.

**Table 1:** General characteristics of studied patients

	Overall	Men				Women				
		All rhythm	SR	AF		All rhythm	SR	AF		
	n = 247	n = 165	n = 28	n = 137	P1	n = 82	n = 35	n = 47	P1	P2
Age, yo	65 [57, 72]	63 [55, 71]**	68 [59, 74]	63 [55, 70]	0.18	69 [62, 73]**	66 [57, 78]	70 [65, 73]	0.34	0.001
Men, n (%)	165/247 (67)									< 0.001
Body mass index, kg/m^2^	25.8 ± 4.1	26.1 ± 3.4	26.1 ± 3.2	26.1 ± 3.5	> 0.99	25.1 ± 5.3	25.1 ± 6.0	25.2 ± 4.7	0.95	0.1
EAT, mL	125.4 ± 47.2	131.8 ± 48.3**	120.9 ± 52.4	134 ± 47.3	0.19	112.5 ± 42.3**	103 ± 42.1	119.6 ± 41.6	0.082	0.002
EATVI, mL/m^2^	71.8 ± 25.7	71.6 ± 25.3	67.4 ± 28.3	72.4 ± 24.6	0.34	72.2 ± 26.6	66.9 ± 27.2	76.1 ± 25.8	0.12	0.87
Atrial fibrillation, n (%)	184/247 (74)	137/165 (83)***	0/28 (0)	137/137 (100)	< 0.001	47/82 (57)***	0/35 (0)	47/47 (100)	< 0.001	< 0.001
Type 2 diabetes mellitus, n (%)	69/247 (28)	52/165 (32)	11/28 (39)	41/137 (30)	0.45	17/82 (21)	10/35 (29)	7/47 (15)	0.22	0.1
Hypertension, n (%)	176/247 (71)	113/165 (69)	19/28 (68)	94/137 (69)	> 0.99	63/82 (77)	29/35 (83)	34/47 (72)	0.39	0.22
Antihypertensive drug, n (%)	193/247 (78)	127/165 (77)	15/28 (54)	112/17 (82)	0.003	66/82 (81)	23/35 (66)	43/47 (92)	0.009	0.64
ACE or ARB, n (%)	109/247 (44)	75/165 (46)	7/28 (25)	68/137 (50)	0.029	34/82 (42)	12/35 (34)	22/47 (47)	0.36	0.65
Calcium blocker, n (%)	101/247 (41)	59/165 (36)*	5/28 (18)	54/137 (39)	0.051	42/82 (51)*	13/35 (37)	29/47 (62)	0.048	0.029
Beta blocker, n (%)	91/247 (37)	69/165 (42)	3/28 (11)	66/137 (48)	0.001	22/82 (27)	3/35 (8.6)	19/47 (40)	0.003	0.031
LVEF, %	66 [61, 71]	65 [60, 71]	63 [60, 66]	66 [60, 72]	0.11	67 [62, 72.8]	65 [62, 69]	69 [63, 74]	0.017	0.062
Left atrial volume, mL	54.9 [42, 71.3]	60 [44, 78]**	53.2 [41.9, 64.2]	61 [45, 78]	0.17	51 [38.4, 60]**	43.2 [35.3, 53.1]	54 [43.5, 62.5]	0.01	0.001
Left atrial volume index, mL/m^2^	32 [24.1, 41.9]	32.4 [24.5, 42.2]	29.5 [22.0, 38]	33.0 [24.3, 42.4]	0.31	31 [26, 38.6]	28 [22.5, 34.5]	35.9 [28.1, 44.1]	0.015	0.71
E/E’	9.0 [7.0, 12.0]	9.0 [7.0, 12.0]*	6.7 [5.3, 7.8]	9.0 [7.0, 12.0]	< 0.001	10.0 [8.0, 12.4]*	8.0 [7.0, 10.4]	11.5 [9.0, 14.0]	< 0.001	0.032

P1 between SR vs. AF in each sex; P2 between all-rhythm men vs. women

#### Men vs. women (all rhythm)

Men who had combined SR and AF (n = 165) were younger than women (Table 1). EATV was larger in men than in women (men, 131.8 ± 48.3 vs. women, 112.5 ± 42.3; P = 0.002); however, EATVI was comparable between men and women. The use of antihypertensive drugs, angiotensin-converting enzyme (ACE) inhibitors, or angiotensin II receptor antagonists (ARB) was similar, while the use of calcium blockers was lower, and the use of beta-blockers was higher in men. The LA volume was higher in men than in women, and the LAVI was comparable between men and women. E/E’ was lower in men than in women.

#### SR vs. AF

In men, age, BMI, EAT, and EATVI were comparable between the SR and AF groups. The prevalence of type 2 diabetes mellitus and hypertension was similar between the SR and AF groups; however, the use of antihypertensive drugs was higher in the AF group. LVEF, LA volume, and LAVI were comparable between the SR and AF groups; however, E/E’ was lower in patients with SR. In women, age, BMI, EAT, and EATVI were similar between the SR and AF groups. The prevalence of type 2 diabetes mellitus and hypertension was comparable between the SR and AF groups; however, the use of antihypertensive drugs was higher in the AF group. LVEF and LAVI were comparable between the SR and AF groups; however, E/E’ was lower in patients with SR.

### Comparison of EATVI and LAVI between SR and AF groups

**Fig. 2:**
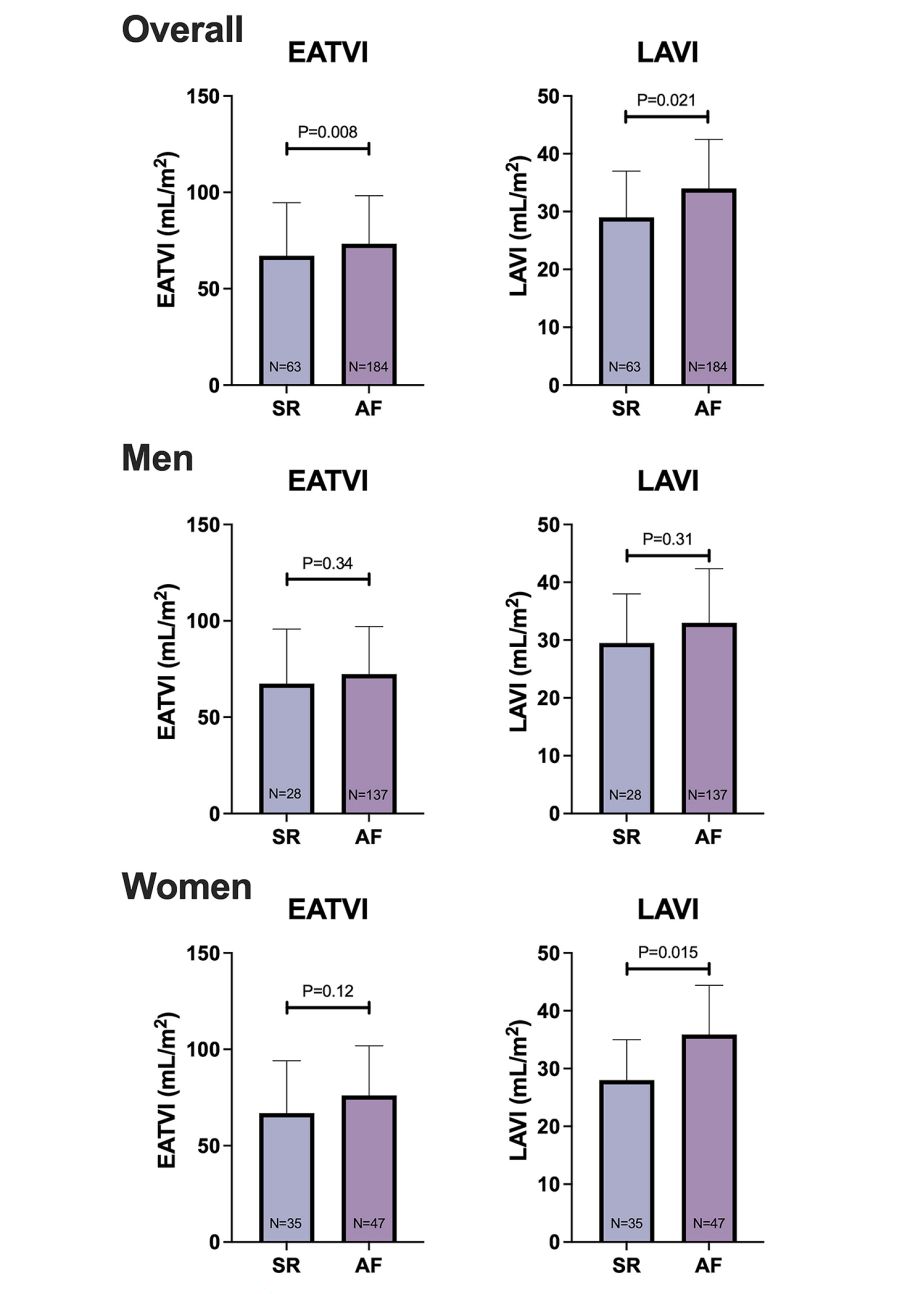
Comparison of EATVI and LAVI between SR and AF in overall, men, and women. Values are presented as mean ± SD. P values were obtained by a two-tailed unpaired t-test. EATVI, epicardial adipose tissue volume index; SR, sinus rhythm; AF, atrial fibrillation; EATV, epicardial adipose tissue volume; LAVI, left atrial volume index

Overall, EATVI and LAVI were higher in the AF group than in the SR group (Fig. 2). In men, EATVI and LAVI were comparable between the SR and AF groups. In women, EATVI was comparable between the SR and AF groups, whereas LAVI was higher in the AF group than in the SR group.

### The relationship between EATVI and LAVI

#### All rhythm

**Fig. 3:**
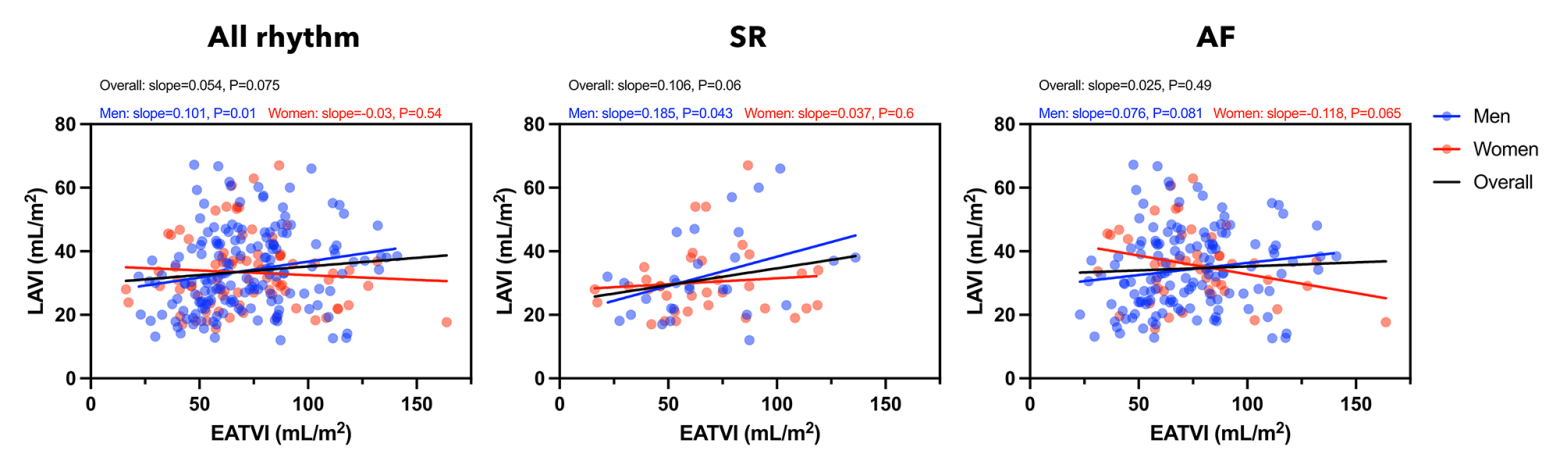
Association between EATVI and LAVI in overall, SR and AF. EATVI, epicardial adipose tissue volume index; LAVI, left atrial volume index; SR, sinus rhythm; AF, atrial fibrillation. Univariate linear regression models showed the relationship between EATVI and LAVI in overall, SR and AF groups. The r and P-values are shown in men (blue circles and lines) and women (red circles and lines)

Univariate analysis showed that EATVI was positively correlated with LAVI in men, but not in women (Fig. 3, left panel).

#### SR

In overall patients, univariate analysis showed that only age was positively correlated with LAVI (Fig. 3, middle panel, and Table 2 A). In men, univariate analysis showed that only EATVI was positively correlated with LAVI (Fig. 3, middle panel, and Table 2 A). Multivariate analysis revealed that EATVI was not associated with LAVI after correcting for confounding factors (Models 1 and 2)(Table 2 A). In women, univariate and multivariate analyses showed that no significant factors were correlated with LAVI (Fig. 2; Table 2).

#### AF

Univariate analysis showed that age and beta-blocker use were positively correlated with LAVI in overall patients (Table 2B). Univariate and multivariate analyses showed no significant correlation between EATVI and LAVI (Fig. 3, right panel, and Table 2B). In men, the univariate analysis showed that the use of antihypertensive drugs was positively correlated with LAVI (Table 2B). Univariate and multivariate analyses showed no significant correlation between the EATVI and LAVI (Fig. 3, right panel). In women, there was a negative correlation between EATVI and LAVI, not significantly, but a trend (Fig. 3, right panel). Univariate analysis showed no significant factors correlated with LAVI (Table 2B). However, multivariate analysis showed that age was positively correlated and EATVI was negatively correlated with LAVI (Models 1 and 2)(Table 2B).

**Table 2:** Linear regression models to determine the factors associated with left atrial volume index

**A. Sinus rhythm**																								
Overall (n = 63)	Univariate									Model 1								Model 2						
Factors	Coefficient	95% CI				P value				Coefficient	95% CI				P value			Coefficient		95% CI			P value	
Age, yo	0.263	0.042		0.485		0.023				0.181	-0.082			0.444	0.18			0.212		-0.054		0.478	0.12	
Men	2.014	-4.083		8.11		0.52				-0.195	-0.841			0.452	0.56			-0.127		-0.778		0.523	0.7	
BMI, kg/m^2^	-0.195	-0.816		0.426		0.54																		
EAT, mL	0.05	-0.014		0.113		0.13																		
EATVI, mL/m^2^	0.106	-0.002		0.214		0.06				0.073	-0.054			0.201	0.27			0.072		-0.057		0.2	0.28	
LVEF, %	-0.385	-0.981		0.211		0.21												-0.457		-1.052		0.138	0.14	
Hypertension, yes	0.565	-6.571		7.7		0.88												-0.594		-7.735		6.548	0.87	
Diabetes mellitus, yes	1.548	-4.889		7.984		0.64																		
Antihypertensive drug, yes	-2.965	-9.133		3.203		0.35																		
ACE or ARB use	-1.447	-8.06		5.166		0.67																		
Beta blocker use	-3.681	-13.994		6.633		0.49																		
Men (n = 28)	Univariate									Model 1								Model 2						
Factors	Coefficient	95% CI				P value				Coefficient	95% CI				P value			Coefficient		95% CI			P value	
Age, yo	0.296	-0.144		0.736		0.2				0.276	-0.188			0.741	0.26			0.219		-0.273		0.711	0.39	
BMI, kg/m^2^	0.86	-0.744		2.464		0.3				0.459	-1.476			2.394	0.65			0.75		-1.265		2.765	0.47	
EAT, mL	0.093	0		0.186		0.061																		
EATVI, mL/m^2^	0.185	0.014		0.355		0.043				0.141	-0.072			0.355	0.21			0.122		-0.096		0.341	0.28	
LVEF, %	-0.845	-1.898		0.207		0.13												-0.697		-1.835		0.442	0.24	
Hypertension, yes	1.071	-9.914		12.056		0.85												-1.261		-12.338		9.816	0.83	
Diabetes mellitus, yes	7.935	-2.125		17.995		0.13																		
Antihypertensive drug, yes	-0.019	-10.313		10.275		1																		
ACE or ARB use	1.529	-10.313		13.37		0.8																		
Beta blocker use	-6.663	-23.063		9.737		0.43																		
Women (n = 35)	Univariate									Model 1								Model 2						
Factors	Coefficient	95% CI				P value				Coefficient	95% CI				P value			Coefficient		95% CI			P value	
Age, yo	0.246	0.002		0.49		0.056				0.306	-0.033			0.646	0.087			0.371		-0.007		0.749	0.064	
BMI, kg/m^2^	-0.463	-1.077		0.152		0.15				-0.316	-0.946			0.313	0.33			-0.259		-0.916		0.397	0.45	
EAT, mL	-0.009	-0.099		0.081		0.85																		
EATVI, mL/m^2^	0.037	-0.101		0.176		0.6				-0.066	-0.248			0.116	0.48			-0.076		-0.264		0.111	0.43	
LVEF, %	-0.05	-0.768		0.668		0.89												-0.331		-1.096		0.435	0.4	
Hypertension, yes	0.928	-8.966		10.822		0.86												-0.642		-10.572		9.289	0.9	
Diabetes mellitus, yes	-4.856	-12.947		3.235		0.25																		
Antihypertensive drug, yes	-5.171	-12.83		2.488		0.19																		
ACEI or ARB use	-3.084	-10.874		4.705		0.44																		
Beta blocker use	-1.023	-14.345		12.3		0.88																		
**B. Atrial fibrillation**																								
Overall (n = 184)	Univariate								Model 1									Model 2						
Factors	Coefficient		95% CI			P value			Coefficient			95% CI			P value			Coefficient	95% CI				P value	
Age, yo	0.23		0.054		0.407	0.011			0.277			0.091	0.464		0.004			0.273	0.084		0.462		0.005	
Men	-1.353		-5.417		2.711	0.51			0.483			-0.016	0.981		0.059			0.347	-0.163		0.858		0.18	
BMI, kg/m^2^	0.299		-0.162		0.76	0.21																		
EAT, mL	0.01		-0.029		0.048	0.63																		
EATVI, mL/m^2^	0.025		-0.046		0.097	0.49			-0.021			-0.098	0.056		0.6			-0.017	-0.093		0.059		0.66	
LVEF, %	-0.134		-0.316		0.047	0.15												-0.167	-0.349		0.014		0.073	
Hypertension, yes	3.718		-0.1		7.536	0.058												2.891	-0.987		6.769		0.15	
Diabetes mellitus, yes	1.802		-2.23		5.834	0.38																		
Antihypertensive drug, yes	5.695		0.897		10.494	0.021																		
ACEI or ARB use	3.327		-0.189		6.843	0.065																		
Beta blocker use	3.72		0.202		7.237	0.04																		
Men (n = 137)	Univariate								Model 1									Model 2						
Factors	Coefficient		95% CI			P value			Coefficient			95% CI			P value			Coefficient	95% CI				P value	
Age, yo	0.205		-0.003		0.413	0.056			0.218			-0.006	0.443		0.059			0.206	-0.021		0.432		0.077	
BMI, kg/m^2^	0.379		-0.228		0.985	0.22			0.418			-0.271	1.107		0.24			0.302	-0.391		0.996		0.39	
EAT, mL	0.033		-0.012		0.077	0.15																		
EATVI, mL/m^2^	0.076		-0.009		0.162	0.081			0.035			-0.063	0.132		0.49			0.036	-0.06		0.133		0.46	
LVEF, %	-0.136		-0.344		0.072	0.2												-0.171	-0.379		0.037		0.11	
Hypertension, yes	4.014		-0.496		8.525	0.083												3.715	-0.871		8.301		0.11	
Diabetes mellitus, yes	2.687		-1.913		7.286	0.25																		
Antihypertensive drug, yes	6.406		1.034		11.779	0.021																		
ACEI or ARB use	3.74		-0.446		7.926	0.082																		
Beta blocker use	4.48		0.312		8.648	0.037																		
Women (n = 47)	Univariate								Model 1									Model 2						
Factors	Coefficient		95% CI			P value			Coefficient			95% CI			P value			Coefficient	95% CI				P value	
Age, yo	0.374		-0.04		0.789	0.084			0.455			0.056	0.855		0.031			0.461	0.048		0.873		0.034	
BMI, kg/m^2^	0.22		-0.472		0.913	0.54			0.46			-0.2	1.119		0.18			0.342	-0.394		1.079		0.37	
EAT, mL	-0.07		-0.146		0.006	0.079																		
EATVI, mL/m^2^	-0.118		-0.24		0.004	0.065			-0.151			-0.272	-0.03		0.018			-0.145	-0.269		-0.021		0.028	
LVEF, %	-0.201		-0.608		0.206	0.34												-0.15	-0.563		0.263		0.48	
Hypertension, yes	2.62		-4.601		9.842	0.48												1.123	-6.141		8.388		0.76	
Diabetes mellitus, yes	-1.029		-10.148		8.091	0.83																		
Antihypertensive drug, yes	0.979		-10.659		12.617	0.87																		
ACEI or ARB use	2.24		-4.237		8.717	0.5																		
Beta blocker use	1.816		-4.782		8.414	0.59																		

ACE or ARB, angiotensin converting enzyme inhibitor or angiotensin receptor blocker; BMI, body mass index; EATV, epicardial adipose tissue volume; EATVI, epicardial adipose tissue volume index; LVEF, left ventricular ejection fraction

### Subgroup analysis

#### Overall

**Fig. 4:**
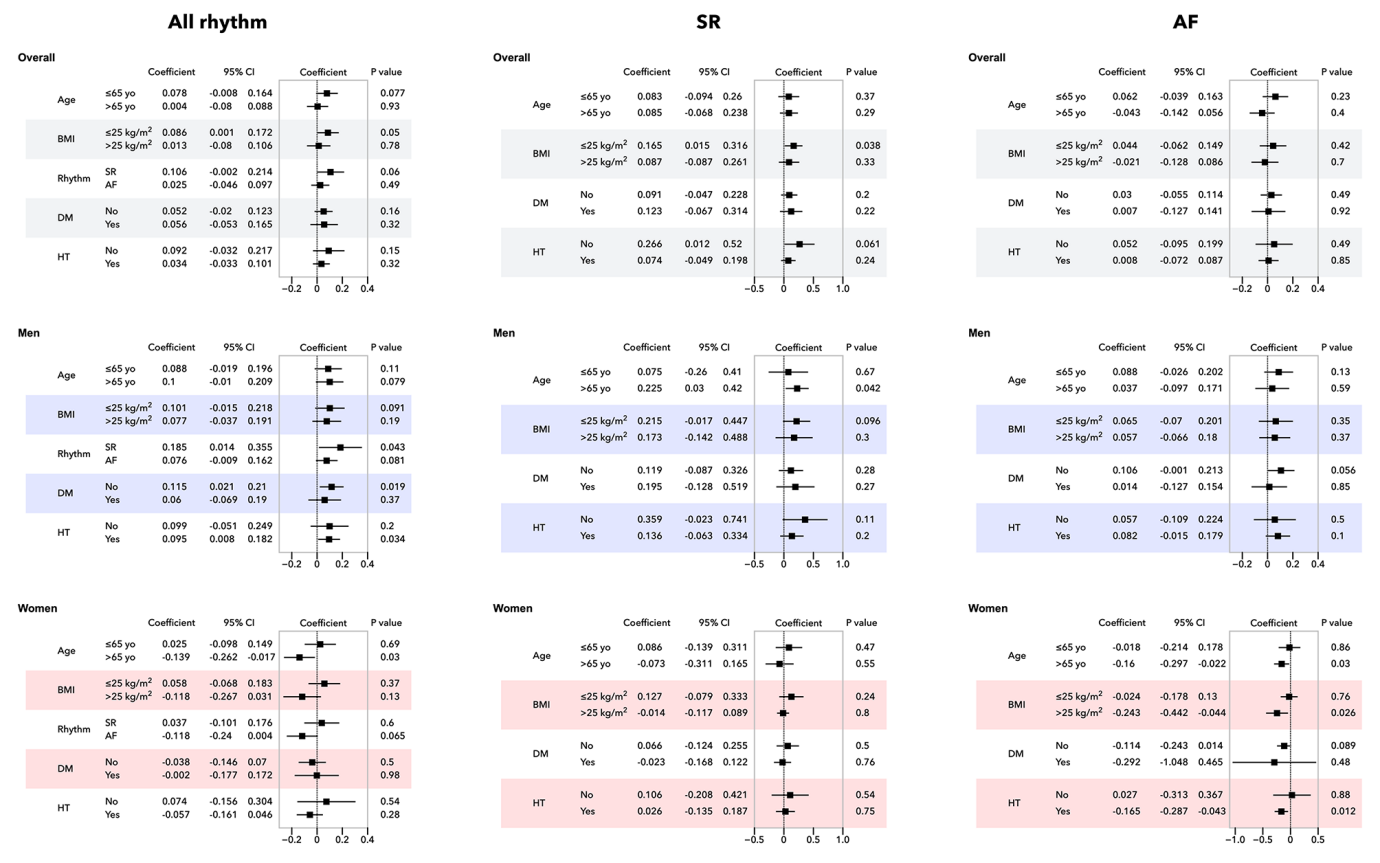
Sub-analysis for the association between EATVI and LAVI in all rhythm, SR and AF. The interaction between men and women for the association between EATVI and LAVI was evaluated using linear regression models for all rhythm, SR, and AF. EATVI, epicardial adipose tissue volume index; LAVI, left atrial volume index; SR, sinus rhythm; AF, atrial fibrillation

EATVI was positively correlated with LAVI for BMI ≤ 25 in patients with SR (Fig. 4, upper row in the column of all rhythm). However, EATVI was not correlated with LAVI for all subgroups in patients with all rhythms or AF (Fig. 4, upper row in the column of SR and AF).

#### Men

In all rhythms (Fig. 4, middle row in the column of all rhythm), EATVI was positively correlated with LAVI in the subgroups of SR, DM (no), and HT (yes). In SR, the EATVI was positively correlated with the LAVI in patients aged ≥ 65 years (Fig. 4, middle row in the column of SR). No significant factors were associated with LAVI in AF (Fig. 4, middle row in the column of AF).

#### Women

In all rhythms (Fig. 4, lower row in the column of all rhythm), EATVI was negatively correlated with LAVI in the subgroup of patients aged > 65 years. There were no significant factors associated with LAVI in SR (Fig. 4, lower row in the column of SR) In AF, EATVI was negatively correlated with LAVI in the subgroups of age > 65 years, BMI > 25, and HT (yes) (Fig. 4, lower row in the column of AF).

## Discussion

In this study, we evaluated sex differences in the association between EATVI and LAVI in patients with either SR or AF, and found two major findings. First, in overall, that includes both men and women, EATVI and LAVI were not significantly correlated with SR and AF. Second, when analyzed separately in men and women, the relationship between EATVI and LAVI differed between men and women. In patients with SR, there was a positive relationship between EATVI and LAVI in men, but not in women. In contrast, in patients with AF, a negative relationship was found between EATVI and LAVI in women, whereas no association was found in men. This is the first report to evaluate sex differences in the relationship between EATV and LAVI, suggesting that the effect of EAT on LAVI may differ between men and women.

### Relationship between EATVI and LAVI in overall patients

EATV has been reported to be associated with the incidence and prevalence of AF [17, 18]. EATV has also been associated with an increased LA size [19]. The prevalence and incidence of AF and LA size are closely and mutually related [9, 19]. In other words, the larger the LA size, the more AF will develop; conversely, when AF develops, LA size will increase [17, 18]. This study showed that EATV and LAVI were not significantly correlated with SR and AF. These results are not consistent with those of previous reports. However, as discussed below, the relationship between EATVI and LAVI was found to be significant when analyzed separately by sex.

### Sex differences in the association between EATVI and LAVI in patients with SR

The relationship between the EATVI and LAVI differed between men and women in both the SR and AF groups. Sex differences in the degree of EATV and its clinical significance have been reported. The relationship between increased EATV and the presence of CAD [1] or a history of coronary artery bypass graft surgery [11] was found in men, but not in women. In our patients with SR, there was a positive relationship between EATVI and LAVI in men, but not in women. Fox et al. showed that in patients with SR, EATV correlated with LA dimension in men but not in women, which is in agreement with our results [19]. Fox et al. [19] and our results support that EATV is involved in LA size or LAVI in men, but not in women.

### Sex differences in the association between EATVI and LAVI in patients with AF

Few studies have reported sex differences in the relationship between EATV and AF. van Rosendael et al. showed that EATV was a factor in the development of AF only in men, even after correcting for AF risk factors [20]. We also reported that EATV was a factor in the development of both PAF and PeAF in men [8]. To our knowledge, this is the first report to examine the association between EATV and LA structure, such as LA size or LAVI, in patients with AF. Our results showed a negative relationship between EATVI and LAVI in women with AF, suggesting that a larger EATVI suppresses the increase in LAVI in women with AF. Because this was a cross-sectional study, cause-and-effect relationships could not be determined. However, the sex difference in EATVI and LAVI in patients with AF is an interesting result and prompts further investigation in future studies.

### Potential mechanisms of sex differences in the relationship between EATVI and LAVI

In men, there was a significant correlation between EATVI and LAVI in the SR group (Table 2). These correlations were found in the SR and DM (no) subgroups for all rhythms and in those aged ≥ 65 years in the SR group (Fig. 4); however, no correlations were found in the AF group. Theoretically, the deleterious effects of EATVI on LA size might be observed more clearly in patients with SR than in those with AF [19], since AF strongly affects LA function and size [21]. In contrast, in women, EATVI was not correlated with LAVI in the SR group but was negatively correlated with LAVI in the AF group (Table 2). Currently, there are no reasonable explanations for this; however, we have attempted to provide hypothetical explanations. In women with AF, EATVI was negatively correlated with LAVI in the subgroups of age > 65 years, BMI > 25, and HT (yes). Therefore, it can be assumed that EATVI inhibits LA enlargement in preobese elderly women. Ovarian estradiol inhibits left ventricular remodeling and protects against LA diastolic dysfunction [22]. However, the present study found a negative link between EATVI and LAVI in menopausal women, indicating effects other than those of ovarian estradiol. There are two possible mechanisms through which EATVI inhibits the increase in LAVI. First, EATVI may be linked to the favorable effects of estradiol and the protective adipocytokine profiles. Estradiol declines rapidly after the loss of ovarian function in menopause; however, it is continuously produced in the subcutaneous (SAT) and visceral adipose tissue (VAT) [23] in EAT [24]. We previously showed that anti-inflammatory adiponectin was largely produced, and proinflammatory IL1B and NLRP3 were less abundant in SAT and VAT of menopausal women than of men [25]. The anti-inflammatory and anti-fibrotic patterns of estradiol and adipocytokines in menopausal women with a larger EATVI could be protective against LA function. However, previous reports are against the protective effects of EAT on cardiac function in menopausal women [26, 27]. Second, sex differences in heart cells, including myocytes, endothelial cells, smooth muscle cells, macrophages, fibroblasts, and valve cells, may be linked to the association between EATVI and LAVI [28]. Quantitative and qualitative differences in the local EAT and whole-body adiposity may differentially affect LA function in men and women. However, the sex differences and underlying mechanisms observed in the current study should be reconfirmed in future studies. If there are sex differences in the effect of EATV on LA size and LA function, it suggests that measures to prevent cardiovascular events related to LA abnormalities need to be considered separately for men and women [29].

### Limitations

First, the cross-sectional design of this study limited the interpretation of causality. Second, the predominantly Japanese patient sample in two recruit location could be biased and limits the generalizability of our findings to a broader population. Third, we did not measure waist circumference or waist-to-hip ratio, which may have added incremental information on local versus systemic adiposity effects. Fourth, AF frequently develops in elderly individuals, who are typically lean. Our study subjects were relatively young and obese, which may have biased the results. Finally, the small subgroup sizes limited the number of adjusted variables in the binary logistic regression models to avoid overfitting the models. Furthermore, the small subgroup sizes could make β error and tend to yield extreme data with no reproducibility. This is the first report on sex differences in EATVI and LAVI, with an exploratory analysis of the hypothesis not performed a priori. This study is not a conclusive design and we should be careful in the interpretation of this finding. Therefore, future large, unbiased and prospective studies, including external validation, are required to address these conclusions and detailed mechanisms.

## Conclusion

We evaluated the sex differences in the association between EATV and LAVI in patients with either SR or AF. We found a positive relationship among men with SR, and a negative relationship among women with AF. This is the first report to evaluate the relationship between EATV and LAVI, divided by sex, and may suggest clinical implications of sex differences in the etiology of AF.

## Data Availability

Derived data supporting the findings of this study are available from the corresponding author on reasonable requests.

## References

[1] Sacks HS, Fain JN. Human epicardial adipose tissue: a review. Am Heart J 2007, 153(6):907–17. 10.1016/j.ahj.2007.03.019 17540190

[2] Shimabukuro M (2009). Cardiac adiposity and global cardiometabolic risk: new concept and clinical implication. Circ J.

[3] Konishi M, Sugiyama S, Sugamura K, Nozaki T, Ohba K, Matsubara J, Matsuzawa Y, Sumida H, Nagayoshi Y, Nakaura T (2010). Association of pericardial fat accumulation rather than abdominal obesity with coronary atherosclerotic plaque formation in patients with suspected coronary artery Disease. Atherosclerosis.

[4] Maimaituxun G, Shimabukuro M, Fukuda D, Yagi S, Hirata Y, Iwase T, Takao S, Matsuura T, Ise T, Kusunose K et al. Local thickness of Epicardial Adipose tissue surrounding the Left Anterior descending artery is a simple predictor of coronary artery Disease- New Prediction Model in Combination with Framingham Risk score. Circ J 2018, 82(5):1369–78. 10.1253/circj.CJ-17-1289 29563352

[5] Shimabukuro M, Hirata Y, Tabata M, Dagvasumberel M, Sato H, Kurobe H, Fukuda D, Soeki T, Kitagawa T, Takanashi S, Sata M (2013). Epicardial adipose tissue volume and adipocytokine imbalance are strongly linked to human coronary Atherosclerosis. Arterioscler Thromb Vasc Biol.

[6] Park HE, Choi SY, Kim M (2014). Association of epicardial fat with left ventricular diastolic function in subjects with metabolic syndrome: assessment using 2-dimensional echocardiography. BMC Cardiovasc Disord.

[7] Fontes-Carvalho R, Fontes-Oliveira M, Sampaio F, Mancio J, Bettencourt N, Teixeira M, Rocha Goncalves F, Gama V, Leite-Moreira A (2014). Influence of epicardial and visceral fat on left ventricular diastolic and systolic functions in patients after Myocardial Infarction. Am J Cardiol.

[8] Oba K, Maeda M, Maimaituxun G, Yamaguchi S, Arasaki O, Fukuda D, Yagi S, Hirata Y, Nishio S, Iwase T et al. Effect of the Epicardial adipose tissue volume on the prevalence of Paroxysmal and Persistent Atrial Fibrillation. Circ J 2018, 82(7):1778–87. 10.1253/circj.CJ-18-0021 29806623

[9] Mahabadi AA, Lehmann N, Kälsch H, Bauer M, Dykun I, Kara K, Moebus S, Jöckel K-H, Erbel R, Möhlenkamp S (2014). Association of epicardial adipose tissue and left atrial size on non-contrast CT with atrial fibrillation: the Heinz Nixdorf Recall Study. Eur Heart J - Cardiovasc Imaging.

[10] Dagvasumberel M, Shimabukuro M, Nishiuchi T, Ueno J, Takao S, Fukuda D, Hirata Y, Kurobe H, Soeki T, Iwase T (2012). Gender disparities in the association between epicardial adipose tissue volume and coronary Atherosclerosis: a 3-dimensional cardiac computed tomography imaging study in Japanese subjects. Cardiovasc Diabetol.

[11] Maimaituxun G, Shimabukuro M, Salim HM, Tabata M, Yuji D, Morimoto Y, Akasaka T, Matsuura T, Yagi S, Fukuda D (2017). Gender-linked impact of epicardial adipose tissue volume in patients who underwent coronary artery bypass graft Surgery or non-coronary valve Surgery. PLoS ONE.

[12] Taylor AJ, Cerqueira M, Hodgson JM, Mark D, Min J, O’Gara P, Rubin GD, ACCF/SCCT/ACR/AHA, ASE/ASNC/NASCI/SCAI/SCMR. 2010 Appropriate Use Criteria for Cardiac Computed Tomography: A Report of the American College of Cardiology Foundation Appropriate Use Criteria Task Force, the Society of Cardiovascular Computed Tomography, the American College of Radiology, the American Heart Association, the American Society of Echocardiography, the American Society of Nuclear Cardiology, the North American Society for Cardiovascular Imaging, the Society for Cardiovascular Angiography and Interventions, and the Society for Cardiovascular Magnetic Resonance. Journal of the American College of Cardiology 2010, 56(22):1864-94. 10.1016/j.jacc.2010.07.005 21087721

[13] Maimaituxun G, Kusunose K, Yamada H, Fukuda D, Yagi S, Torii Y, Yamada N, Soeki T, Masuzaki H, Sata M, Shimabukuro M. Deleterious effects of Epicardial Adipose tissue volume on Global Longitudinal Strain in patients with preserved left ventricular ejection Fraction. Front Cardiovasc Med 2021, 7. 10.3389/fcvm.2020.607825 PMC7843424 33521062

[14] Maimaituxun G, Yamada H, Fukuda D, Yagi S, Kusunose K, Hirata Y, Nishio S, Soeki T, Masuzaki H, Sata M, Shimabukuro M (2020). Association of Local Epicardial Adipose Tissue Depots and left ventricular diastolic performance in patients with preserved left ventricular ejection Fraction. Circ J.

[15] Nagueh SF, Smiseth OA, Appleton CP, Byrd BF, Dokainish H, Edvardsen T, Flachskampf FA, Gillebert TC, Klein AL, Lancellotti P (2016). Recommendations for the evaluation of left ventricular diastolic function by Echocardiography: an update from the American Society of Echocardiography and the European Association of Cardiovascular Imaging. J Am Soc Echocardiogr.

[16] Lang RM, Badano LP, Mor-Avi V, Afilalo J, Armstrong A, Ernande L, Flachskampf FA, Foster E, Goldstein SA, Kuznetsova T (2015). Recommendations for cardiac chamber quantification by echocardiography in adults: an update from the American Society of Echocardiography and the European Association of Cardiovascular Imaging. J Am Soc Echocardiogr.

[17] Al-Rawahi M, Proietti R, Thanassoulis G (2015). Pericardial fat and atrial fibrillation: Epidemiology, mechanisms and interventions. Int J Cardiol.

[18] Wong CX, Ganesan AN, Selvanayagam JB. Epicardial fat and atrial fibrillation: current evidence, potential mechanisms, clinical implications, and future directions. Eur Heart J 2017, 38(17):1294–302. 10.1093/eurheartj/ehw045 26935271

[19] Fox CS, Gona P, Hoffmann U, Porter SA, Salton CJ, Massaro JM, Levy D, Larson MG, D’Agostino RB, Sr., O’Donnell CJ, Manning WJ. Pericardial fat, intrathoracic fat, and measures of left ventricular structure and function: the Framingham Heart Study. Circulation 2009, 119(12):1586-91. 10.1161/CIRCULATIONAHA.108.828970 PMC2727456 19289634

[20] van Rosendael AR, Dimitriu-Leen AC, van Rosendael PJ, Leung M, Smit JM, Saraste A, Knuuti J, van der Geest RJ, van der Arend BW, van Zwet EW et al. Association between posterior left atrial adipose tissue Mass and Atrial Fibrillation. Circ Arrhythm Electrophysiol 2017, 10(2). 10.1161/CIRCEP.116.004614 28183844

[21] Sanfilippo AJ, Abascal VM, Sheehan M, Oertel LB, Harrigan P, Hughes RA, Weyman AE. Atrial enlargement as a consequence of atrial fibrillation. A prospective echocardiographic study. Circulation 1990, 82(3):792–7. 10.1161/01.cir.82.3.792 2144217

[22] Medzikovic L, Aryan L, Eghbali M (2019). Connecting sex differences, estrogen signaling, and microRNAs in cardiac fibrosis. J Mol Med (Berl).

[23] Hetemaki N, Savolainen-Peltonen H, Tikkanen MJ, Wang F, Paatela H, Hamalainen E, Turpeinen U, Haanpaa M, Vihma V, Mikkola TS (2017). Estrogen Metabolism in Abdominal Subcutaneous and visceral adipose tissue in Postmenopausal Women. J Clin Endocrinol Metab.

[24] El Khoudary SR, Shields KJ, Janssen I, Hanley C, Budoff MJ, Barinas-Mitchell E, Everson-Rose SA, Powell LH, Matthews KA (2015). Cardiovascular Fat, Menopause, and sex hormones in women: the SWAN Cardiovascular Fat Ancillary Study. J Clin Endocrinol Metab.

[25] Shimabukuro M, Sato H, Izaki H, Fukuda D, Uematsu E, Hirata Y, Yagi S, Soeki T, Sakaue H, Kanayama HO et al. Depot- and gender-specific expression of NLRP3 inflammasome and toll-like receptors in adipose tissue of cancer patients. Biofactors 2016, 42(4):397–406. 10.1002/biof.1287 27086574

[26] Kim SA, Kim MN, Shim WJ, Park SM. Epicardial adipose tissue is related to cardiac function in elderly women, but not in men. Nutr Metab Cardiovasc Dis 2017, 27(1):41–7. 10.1016/j.numecd.2016.11.001 27988072

[27] Kim JS, Shin SY, Kang JH, Yong HS, Na JO, Choi CU, Kim SH, Kim EJ, Rha SW, Park CG et al. Influence of sex on the Association between Epicardial Adipose Tissue and left atrial transport function in patients with Atrial Fibrillation: a multislice computed Tomography Study. J Am Heart Assoc 2017, 6(8). 10.1161/JAHA.117.006077 PMC5586448 28778939

[28] Walker CJ, Schroeder ME, Aguado BA, Anseth KS, Leinwand LA (2021). Matters of the heart: Cellular sex differences. J Mol Cell Cardiol.

[29] Zheng R, Kusunose K. Linkage of left atrial function to Heart Failure with preserved ejection fraction. Int Heart J 2023, 64(1):4–9. 10.1536/ihj.22-659 36682772

